# Supplementary material for: RNA replication-independent, DNA linearization-dependent expression of reporter genes from a SARS-CoV-2 replicon-encoding DNA in human cells
Source: PLoS One. 2024 Aug 16;19(8):e0300491. doi: 10.1371/journal.pone.0300491 (PMC11329111; doi:10.1371/journal.pone.0300491)

Fig S3. Fluorescence microscopy analysis of replicon RNA-transfected HEK293T cells transduced or not with SARS-CoV-2 N

HEK293T, replicon RNA electroporation

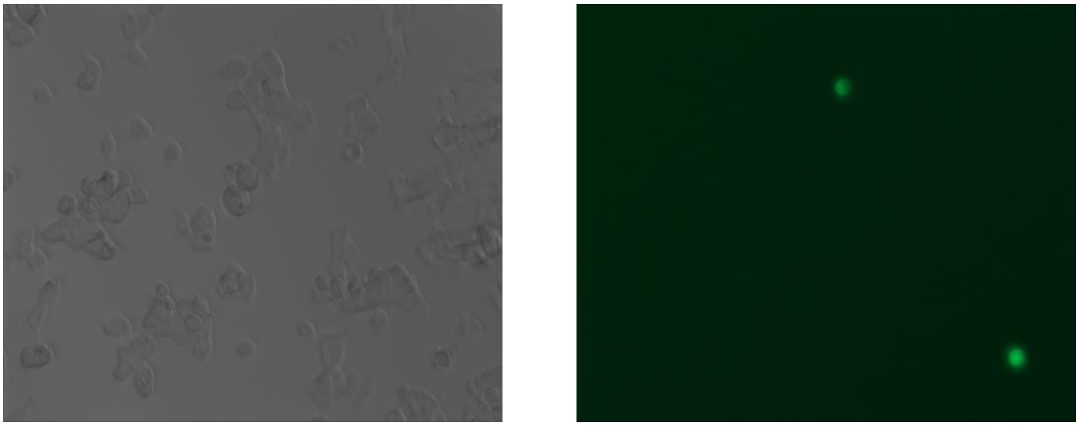

N-transduced HEK293T, replicon RNA electroporation

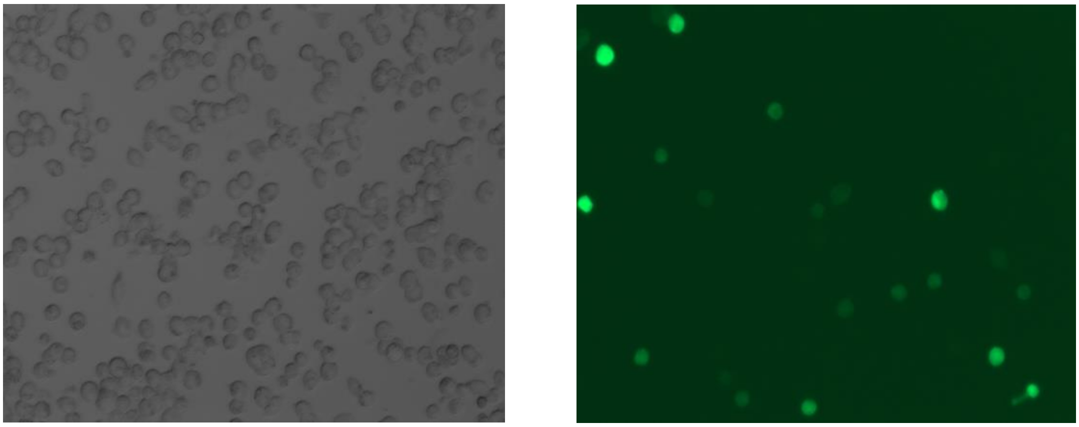

Supplement: S3 Fig — (PDF) [file pone.0300491.s003.pdf]
