# Supplementary material for: RNA replication-independent, DNA linearization-dependent expression of reporter genes from a SARS-CoV-2 replicon-encoding DNA in human cells
Source: PLoS One. 2024 Aug 16;19(8):e0300491. doi: 10.1371/journal.pone.0300491 (PMC11329111; doi:10.1371/journal.pone.0300491)

Fig S4. GFP expression in BAC DNA-transfected Calu-3 cells

Calu3, linearized BAC DNA electroporation

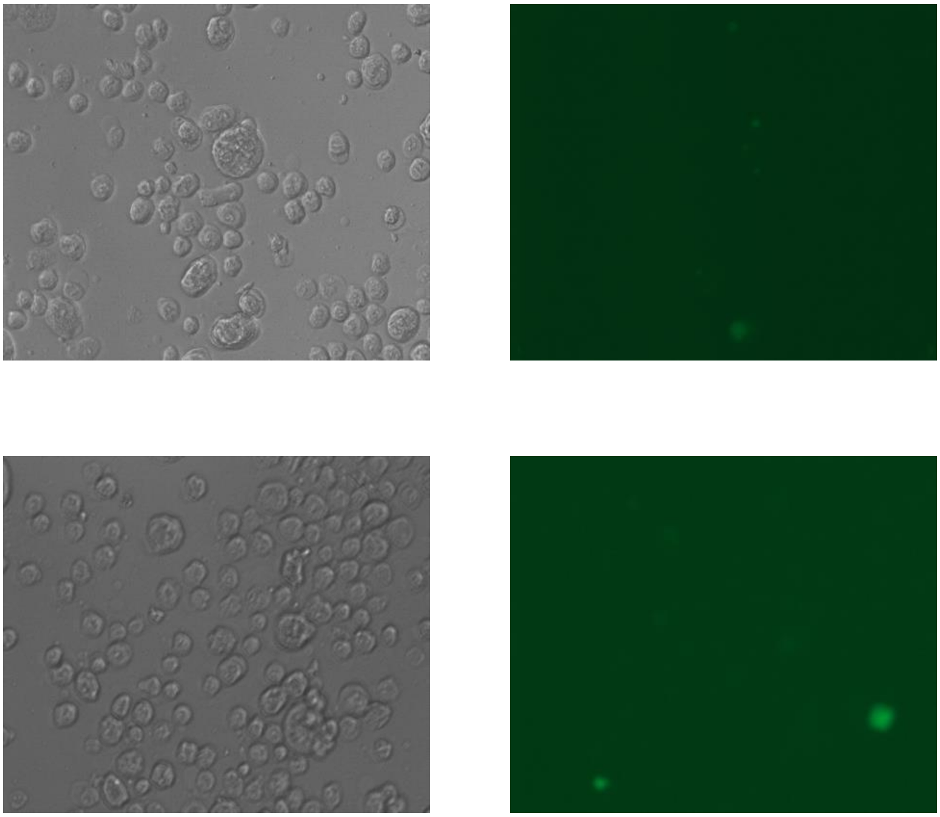

Supplement: S4 Fig — (PDF) [file pone.0300491.s004.pdf]
